# Community-based health screening and all-cause mortality in rural South Africa: a longitudinal cohort study

**DOI:** 10.1136/bmjph-2025-003803

**Published:** 2026-05-28

**Authors:** Faith Magut, Stephen Olivier, Ariane Sessego, Lusanda Mazibuko, Jacob Busang, Emily Wong, Willem Hanekom, Dickman Gareta, Kobus Herbst, Kathy Baisley, Mark Siedner

**Affiliations:** 1 Africa Health Research Institute, Durban, KwaZulu-Natal, South Africa; 2 London School of Hygiene & Tropical Medicine, London, UK; 3 UCL, London, UK; 4 University of Alabama at Birmingham Health System, Birmingham, Alabama, USA; 5 University of Bern, Bern, Switzerland; 6 Medical Practice Evaluation Center, Massachusetts General Hospital, Boston, Massachusetts, USA

**Keywords:** Incidence, Community Participation, Causality, Program Evaluation

## Abstract

**Introduction:**

South Africa is experiencing a significant shift in its disease burden from high HIV and tuberculosis (TB) mortality to increased burden of non-communicable diseases (NCDs). Despite this, screening, treatment and control for NCDs remain limited. Community health fairs are often used to identify and refer individuals with chronic diseases in underserved areas, but their impact on morbidity and mortality is unclear.

**Methods:**

From 2018 to 2020, individuals aged 15+ in the Africa Health Research Institute’s demographic surveillance area in rural KwaZulu-Natal were invited to Vukuzazi, a community health fair programme offering screening and referrals for HIV, TB, hypertension and diabetes. All residents were followed through regular household surveillance to identify deaths. We used inverse probability of treatment weighting survival analysis to estimate the effect of attendance on all-cause mortality and logistic regression to assess whether any difference in mortality may be explained by increased access to care.

**Results:**

Of those eligible, 18 041 (50.0%) participated in Vukuzazi. Individuals were observed after the health fairs for a median of 4.0 years (IQR 3.7–4.2 years) comprising a total of 127 625 person years. Vukuzazi health fair attendance was associated with a 25% relative reduction in the hazard of all-cause mortality (HR=0.75, 95% CI 0.67 to 0.84), corresponding to a 1.5% absolute reduction in mortality over 5 years. In the 3 months post health fair, attenders had 13% increased odds of clinic attendance (OR=1.13, 95% CI 1.10 to 1.17), but no change in clinic attendance among non-attenders (OR=1.02, 95% CI 0.98 to 1.06).

**Conclusion:**

Participation in a community-based health fair was associated with a reduction in 5-year all-cause mortality in rural South Africa. The reduced mortality in those attending corresponded to an increase in primary healthcare engagement following the health fairs. The integration of health fairs with a paired referral system may be an effective strategy to improve health outcomes in the region.

**WHAT IS ALREADY KNOWN ON THIS TOPIC**

- Community-based health screening programmes can improve detection of chronic and infectious diseases and improve linkage to care. However, evidence on whether participation in such programmes influences long-term outcomes such as all-cause mortality remains limited.

**WHAT THIS STUDY ADDS**

- This study examines the association between attendance at community-based health fairs and all-cause mortality. We found a 25% relative risk reduction in all-cause mortality after attendance at such health fairs in rural South Africa.

**HOW THIS STUDY MIGHT AFFECT RESEARCH, PRACTICE OR POLICY**

- These findings highlight the potential value of community-based health fairs for engaging populations in screening and may inform the design and scale-up of population health programmes aimed at improving early diagnosis, engagement in care and long-term health outcomes.

## Introduction

 Since the massive scale-up of the national antiretroviral (ART) programme in 2004, mortality rates in South Africa (SA) have dramatically declined and adult life expectancy has increased.^1^ For example, in KwaZulu-Natal, HIV-related mortality decreased from 56% to 39%^2 3^ and life expectancy increased from 49 years in 2004 to 61 years in 2021.^4^

In the wake of this substantial decline in infectious disease-related deaths, there has been a growth in non-communicable disease (NCD) morbidity and mortality—with over 60% of all deaths in SA now due to NCDs.^5^ However, unlike the massive global investments in HIV and tuberculosis (TB) control, similar outlays into health systems and responses from NCDs have been much less substantial. Moreover, healthcare access and disease control for NCDs such as hypertension and diabetes are substantially lower than they are for HIV,^6 7^ making diabetes, stroke and heart disease the leading causes of death in the region.^8^

Consequently, strategies that target improved healthcare access for NCDs are urgently needed in the SA region. A major priority of the SA Department of Health is to promote the self-empowerment of individuals to improve their health and provide community-based healthcare through non-traditional clinical care delivery, such as community health worker-supported care.^9 10^ One aspect of such community-based approaches includes mobile health clinics and health fairs. Community health fairs are targeted outreach events that offer participants medical screening, vaccination, provision of basic preventive medication, education about positive health behaviours, and referral for linkage to care, as appropriate.^11^,^13^ Health fairs can also guide the allocation of resources in the communities by identifying priorities in terms of highly prevalent conditions or those with poor disease control,^12 14 15^ improve health equity by democratising access to remote areas^16 17^ and provide a communication bridge between community members and the healthcare system.^14^

Yet, despite the many purported benefits of community health fairs, there is a paucity of literature on their direct impacts on health. We sought to estimate the potential benefits of health fair attendance through analysis of data from the Vukuzazi (meaning ‘Wake up and know yourself’ in isiZulu) programme,^18^ which provided a natural experiment to estimate the impact of health fairs on all-cause mortality. The Vukuzazi programme is a large community health fair initiative implemented within the Africa Health Research Institute Health and Demographic Surveillance System (HDSS) in rural KwaZulu-Natal, South Africa. The programme was designed to characterise health through community health fairs delivered via mobile health camps that provided integrated screening for infectious diseases, including HIV and TB, as well as non-communicable diseases such as hypertension, diabetes and obesity. Individuals identified with previously undiagnosed conditions were referred to local primary healthcare facilities for further management. The programme also aimed to characterise the overlapping burden of HIV, TB and non-communicable diseases in this population.

In this study, we aimed to investigate whether attendance at the Vukuzazi community health fairs was associated with reduced all-cause mortality among individuals residing within the HDSS area, and whether attenders had higher presentation to primary healthcare clinics following referral compared with non-attenders. By leveraging longitudinal HDSS surveillance data, this study provides evidence on whether community-based health fairs are associated with improved health outcomes, specifically increased clinic attendance and reduced all-cause mortality.

## Methods

### Study design

This was a nested, prospective cohort study of residents aged 15 years and above in the Africa Health Research Institute HDSS area in the uMkhanyakude District of KwaZulu-Natal. Full details of the HDSS programme have been described previously.^19^

Within the HDSS, a region-wide series of population-based health fairs (Vukuzazi) was conducted May 2018 and March 2020. All resident individuals aged 15 years and older in the HDSS were visited at their homes and invited to participate in the community health fair programme, which were scheduled throughout the southern area of the HDSS catchment area in such a way that all households were within two kilometres of the nearest event.^6^ At the health fair, individuals were screened for HIV, TB, obesity, diabetes and hypertension as well as assessment of lifestyle factors such as smoking and alcohol use. As part of this programme, those newly diagnosed with any of these conditions were referred to the healthcare system for further care.^3 6^ By leveraging the existing HDSS platform, we monitored both clinic presentations and deaths among residents following the health fair.

### Data collection

Full details of the Vukuzazi health fairs have been described previously.^18 20^ Briefly, individuals were interviewed by study nurses about their health status, healthcare access, smoking history and alcohol use. Anthropometric and blood pressure measurements were done using the WHO STEPs protocol.^21^ Blood samples were collected for measurement of glycosylated haemoglobin (glycated haemoglobin (HbA_1c_), measured using the VARIANT II TURBO Haemoglobin testing system (Bio-Rad, Marnes-la-Coquette, Paris, France)) and for HIV (Genscreen Ultra HIV Ag-Ab enzyme immunoassay (Bio-Rad)). Participants with a positive HIV immunoassay had a reflex HIV-1 RNA viral load test done (Abbott RealTime HIV-1 Viral Load (Abbott, Illinois, USA)). All participants underwent sputum collection for TB culture and GeneXpert MTB/RIF Ultra testing; and non-pregnant participants were additionally screened for TB using digital chest X-rays. Participants with new diagnoses, including elevated blood pressure in someone not on antihypertensive treatment, an elevated HbA1c in someone not on diabetes treatment, an X-ray or sputum GeneXpert suggestive of TB, or a new HIV diagnosis were scheduled for a home-based follow-up visit conducted by a study nurse. During this visit, confirmation testing was performed (where applicable) and referrals were made to local primary care clinics. When individuals presented to clinic, a nurse, with access to the health fair testing results, was alerted.^6^

After the health fairs, leveraging the HDSS data, we observed all individuals, including both attenders and non-attenders, longitudinally for presentation to healthcare centres in the catchment area and all-cause mortality. Data on outcomes and covariates for both attenders and non-attenders were obtained through the routine HDSS surveillance. Written informed consent obtained at the health fair applied specifically to participation in the health fair-based activities, while participation in the HDSS surveillance is conducted under its informed consent procedures. In brief, as part of this surveillance, all households are surveyed three times per year to collect data on births, deaths and health status.^19^

All deaths are verified by home-based follow-up verbal autopsy interview.^22^ Moreover, attendance at any of the 11 primary care clinics or hospitalisation at the single public hospital located within the surveillance area is recorded and linked to the HDSS database.

## Measures

### Outcome definition

Our primary outcome was all-cause mortality, as captured by the HDSS. We ascertained all deaths in the HDSS surveillance programme from May 2018 (ie, the start of the Vukuzazi health fairs) through July 2023.

### Exposure definition

Our primary exposure was attendance at a Vukuzazi health fair. Those who presented to the health fairs and consented to data collection were categorised as Vukuzazi attenders. Those who were eligible for the programme but did not attend the health fairs, including those who declined, never presented to the health fairs, or lacked capacity to attend, were classified as non-attenders. Non-attenders to health fairs were assigned health fair dates on the day that the health fair was closest to their residence and eligible for the outcome following that date.

### Confounding variables

We constructed a directed acyclic graph (DAG) to conceptualise the causal relationship between participation in the Vukuzazi programme and all-cause mortality using Dagitty (online supplemental figure 1).^23^ The DAG was used to identify confounders to be considered to maximise the causal inferences drawn from our models of the relationship between health fair attendance and mortality.^24^ Age, sex, educational attainment, employment, household socio-economic status, geographic location defined as rural, urban or peri-urban, and prior healthcare access/seeking, as defined by the recording of any primary care clinic visits in the year prior to the Vukuzazi health fair date, were identified as potential confounders. The socioeconomic status (SES) of the participants was determined using principal component analysis based on ownership of household assets and characteristics such as access to piped water, type of toilet, electricity and type of cooking fuel.^25^

### Statistical analyses

We first examined differences in the characteristics of individuals who attended the Vukuzazi health fairs versus those who did not. We then estimated crude all-cause mortality for each group, with person-time defined from the date of the scheduled Vukuzazi health fair until the earliest of: (1) date of death for those who died; (2) date of migration out of the Africa Health Research Institute (AHRI) demographic surveillance area for those who out-migrated; or (3) date last observed in the surveillance area. We estimated mortality rates for the entire cohort and for age, sex and Vukuzazi-attendance strata.

We estimated the effect of Vukuzazi attendance on all-cause mortality using the inverse probability of treatment weighting (IPTW) survival analysis as the primary analysis approach. The IPTW approach applies a re-weighting procedure of exposed or unexposed individuals based on their characteristics in terms of the potential confounders identified in the DAG. This approach creates a representative pseudo-population where the confounders are independent of the exposure measure, thus eliminating confounding.^26^

To do so, we first estimated the probability of attendance (propensity score) using a logistic regression model with Vukuzazi attendance as the outcome and the potential confounder variables as predictors (online supplemental figure 2). This provided a propensity score (probability of attendance) for each individual in the cohort and we used the propensity scores to generate the inverse probability of treatment weights. The positivity assumption requires that every individual in the study population has a non-zero probability of receiving each treatment or exposure given their characteristics.^27^ To assess the positivity assumption, we observed the overlap in the propensity score between attenders and non-attenders using histogram plots. We also assessed the balance in the covariates between Vukuzazi attenders and non-attenders both before and after IPTW using the standardised mean difference (SMD), with a threshold of an absolute SMD value of 10% indicating covariate balance (online supplemental figure 3).

We then used Kaplan-Meier plots to compare the survival probability and the mean survival time between those who attended Vukuzazi and those who did not, both before and after applying the IPTW weights. We estimated the absolute effect of Vukuzazi attendance on the probability of the occurrence of mortality within the given duration of follow-up. We employed Cox proportional hazard models to estimate the hazard ratios (HRs) while adjusting for the IPTW weights. Schoenfeld residuals were used to assess if the proportional hazard assumption was met. A robust sandwich variance estimator was used to account for the weighted nature of the sample and the randomness in the weights. The HRs were estimated for the overall sample and stratified by age and sex. We used HRs and their respective 95% CIs to quantify the effect of the attendance on all-cause mortality.

We next summarised the causes of death. The causes of death were determined by InterVA 5 from next of kin verbal autopsy interviews.^28^ To assess the robustness of our findings, we conducted a sensitivity analysis where we excluded deaths due to external factors that would not be expected to be prevented due to health fair attendance, such as assaults, road traffic accidents, accidental falls, accidental drownings and submersions, exposure to forces of nature and deaths due to accidental poisoning and noxious substances.

Finally, to explore the mechanism of effect, we compared clinic attendance before versus after the Vukuzazi health fair dates. To observe long-term attendance rates, we graphically depicted clinic visitation in the 12 months before and 12 months after Vukuzazi for both attenders and non-attenders. To determine if Vukuzazi referrals had an immediate effect on healthcare access, we fit generalised estimating equations logistic regression model to estimate whether clinic visit attendance in the 3 months before versus 3 months after the health fairs differed by attendance status. We conducted a sensitivity analysis in which we excluded the COVID-19 period from this analysis (ie, visit dates after 1 March 2020) and conducted sub-analyses by age and sex to determine if clinic visitation changes reflected changes in mortality by sub-groups.

### Patient and public involvement

Participants were not involved in the design of the study, the identification of relevant outcomes, the interpretation of findings or the review of the manuscript. The study results will be disseminated to participants and the HDSS area through roadshows led by the AHRI Community Engagement Unit.

## Results

### Characteristics of the study participants

A total of 36 097 HDSS residents 15 years or older were eligible for participation in the Vukuzazi health fair programme. Of these, 18 041 (50.0% of those eligible) participated in the programme. When compared with non-attenders, Vukuzazi attenders were more likely to be women (68% vs 49%), older (median age 33 vs 31 years), more likely to have primary highest level of education (34% vs 20%), less likely to be employed (20% vs 37%), more likely to have a clinic visit in the past 1 month (53% vs 33%), more likely to have participated in prior HDSS surveys (61% vs 36%), more likely to reside in rural versus peri-urban areas (64% vs 55%), more likely to have a prior positive HIV test in the HDSS (28% vs 18%) and less likely to have out-migrated in the past 5 years (13% vs 21%) (table 1).

**Table 1:** Demographics of population by Vukuzazi attendance

Characteristic	Overall, n=36 097^*^	Did not attend, n=18 056^*^	Attended, n=18 041^*^	P value^†^
Sex (n, %)				<0.001
Male	14 966 (41%)	9154 (51%)	5812 (32%)	
Female	21 131 (59%)	8902 (49%)	12 229 (68%)	
Age (median, IQR)	33 (22, 50)	31 (21, 44)	37 (23, 56)	<0.001
Age group (n, %)				<0.001
15–24	11 015 (31%)	6049 (34%)	4966 (28%)	
25–44	13 610 (38%)	7600 (42%)	6010 (33%)	
45–64	7880 (22%)	3290 (18%)	4590 (25%)	
65+	3592 (10.0%)	1117 (6.2%)	2475 (14%)	
Highest education level (n, %)				<0.001
Primary	9465 (27%)	3493 (20%)	5972 (34%)	
Secondary	22 850 (66%)	12 132 (70%)	10 718 (62%)	
NTC (certificate)	1397 (4.0%)	872 (5.0%)	525 (3.0%)	
College/university	982 (2.8%)	776 (4.5%)	206 (1.2%)	
Missing	1403	783	620	
Employed (n, %)				<0.001
No	23 839 (72%)	10 349 (63%)	13 490 (80%)	
Yes	9502 (28%)	6130 (37%)	3372 (20%)	
Missing	2756	1577	1179	
Any clinic visits in the past year (n, %)				<0.001
No	20 596 (57%)	12 116 (67%)	8480 (47%)	
Yes	15 501 (43%)	5940 (33%)	9561 (53%)	
Participated in the AHRI HDSS survey in the past year (n, %)				<0.001
No	18 652 (52%)	11 598 (64%)	7054 (39%)	
Yes	17 445 (48%)	6458 (36%)	10 987 (61%)	
Socio-economic status (n, %)				<0.001
Lowest	3724 (10%)	1606 (8.9%)	2118 (12%)	
Low	8439 (23%)	3727 (21%)	4712 (26%)	
Middle	7932 (22%)	3716 (21%)	4216 (23%)	
High	6237 (17%)	3122 (17%)	3115 (17%)	
Highest	8140 (23%)	4833 (27%)	3307 (18%)	
(Missing)	1625 (4.5%)	1052 (5.8%)	573 (3.2%)	
Location (n, %)				<0.001
Rural	21 431 (59%)	9941 (55%)	11 490 (64%)	
Peri-urban	11 817 (33%)	6216 (34%)	5601 (31%)	
Urban	2849 (8%)	1899 (11%)	950 (5%)	
KM to nearest clinic (median, IQR)	2.42 (1.41, 3.85)	2.26 (1.31, 3.61)	2.63 (1.52, 4.07)	<0.001
Missing	81	46	35	
Out-migrated in past 5 years (n, %)				<0.001
No	30 030 (83%)	14 312 (79%)	15 718 (87%)	
Yes	6029 (17%)	3714 (21%)	2315 (13%)	
Missing	38	30	8	
Ever tested HIV+ in serosurvey (n, %)				<0.001
No	27 780 (77%)	14 839 (82%)	12 941 (72%)	
Yes	8317 (23%)	3217 (18%)	5100 (28%)	

* n (%); median (IQR).

† Pearson’s χ2 test; Wilcoxon rank sum test.

AHRI, Africa Health Research Institute; HDSS, Health and Demographic Surveillance System; KM, kilometres; NTC, National Technical Certificate.

### Effect of Vukuzazi attendance on mortality

**Figure 1:**
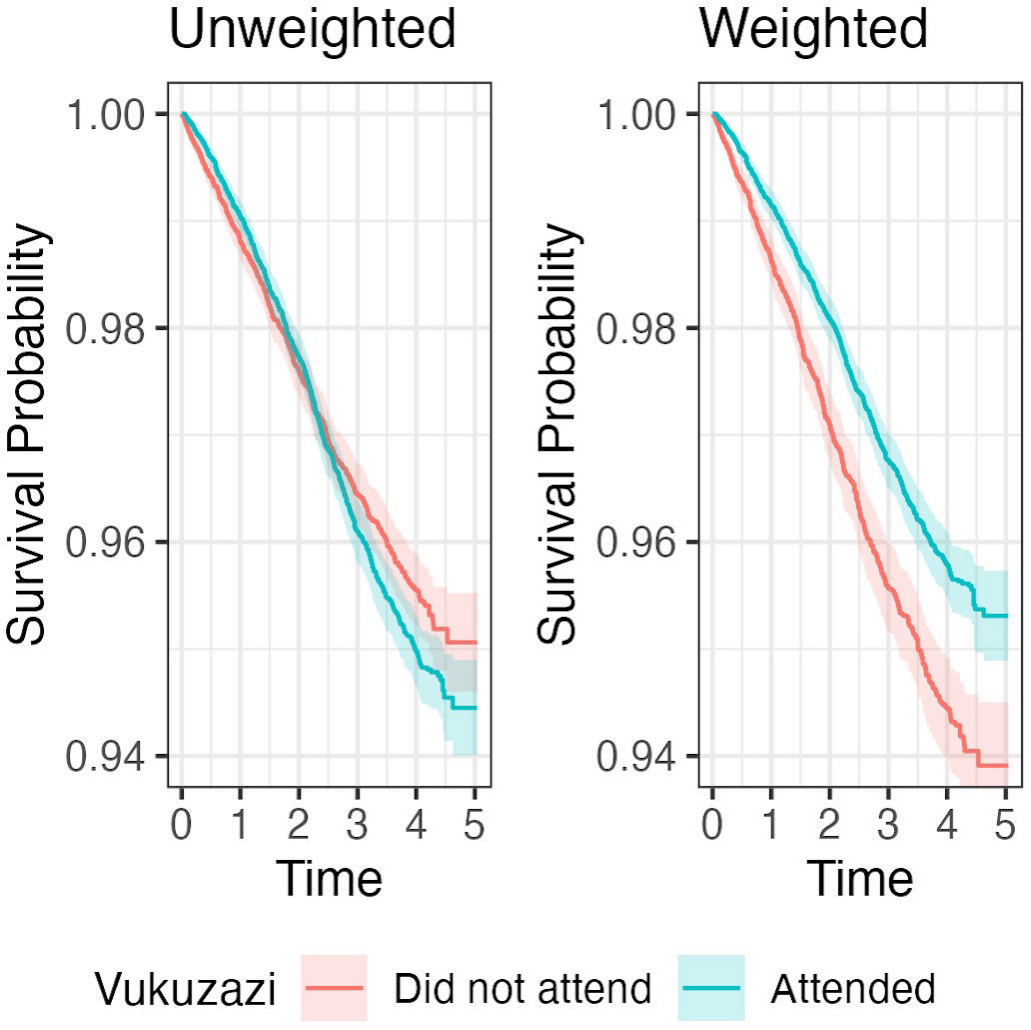
Kaplan-Meier curves of crude survival (weighted and unweighted) in attenders and non-attenders.

Individuals were observed for a median of 4.0 years (IQR 3.7–4.2 years) from the time of their Vukuzazi health fair date until death or data extraction in July 2023, comprising a total of 127 625 person years of observation. A total of 1549 deaths were recorded in the cohort during follow-up time. The crude all-cause mortality rate was 12.14 per 1000 person-years (95% CI 11.54 to 12.76, (online supplemental table 1). After applying probability weights for attendance, there was a significant reduction in mortality in Vukuzazi health fair attenders compared with non-attenders (figure 1), corresponding to an absolute 1.4% reduction in the probability of death within 5 years of follow-up. Health fair attendance was associated with a 27% reduction in the hazard of all-cause mortality (HR: 0.73; 95% CI 0.66 to 0.82) (table 2)

**Table 2:** Effect of Vukuzazi attendance on all-cause mortality

	Mortality	Unadjusted model	IPTW adjusted model
	**n/N(%**)	**HR (95% CI**)	**HR (95% CI**)
Primary analysis			
Attended Vukuzazi			
No	675/16055 (4.2%)	Ref	Ref
Yes	874/17954 (4.9%)	1.12 (1.01 to 1.23)	0.73 (0.66 to 0.82)
Sensitivity analysis			
Attended Vukuzazi			
No	615/15995 (3.8%)	Ref	Ref
Yes	826/17906 (4.6%)	1.16 (1.04 to 1.28)	0.72 (0.64 to 0.81)

IPTW, inverse probability of treatment weighting.

**Figure 2:**
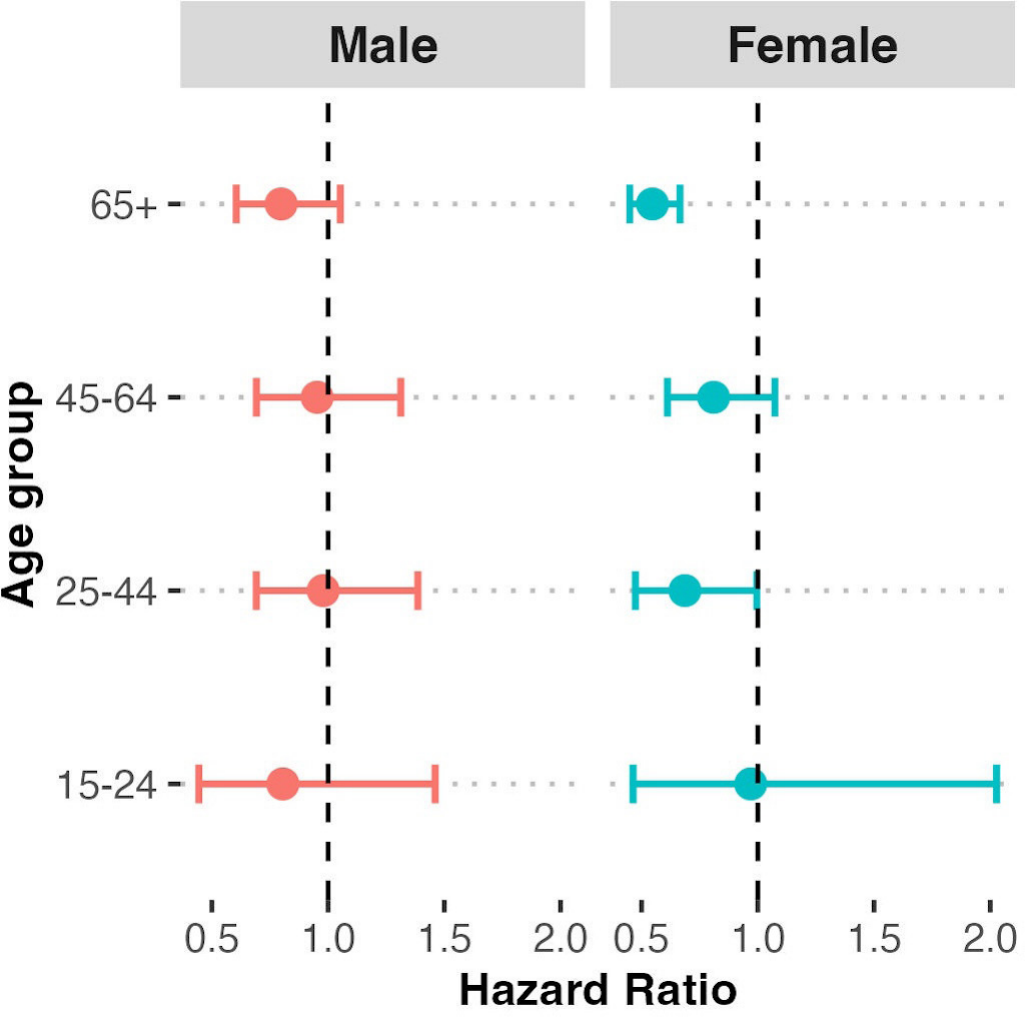
Stratified analysis by age and sex.

In stratified analyses, the benefit of health fair attendance appeared to be greatest in women over 65 years old (figure 2). For example, the hazard of all-cause mortality was 0.55 (95%CI 0.45 to 0.67) for women over 65 and 0.80 (95%CI 0.60 to 1.05) for men over 65; compared with 0.97 (95%CI 0.46 to 2.02) and 0.81 (95%CI 0.44 to 1.46) for women and men 15–24 years old, respectively.

A summary of causes of death, overall and by health fair attendance is summarised in online supplemental table 2. Findings were similar to those of the primary analysis after excluding causes of death due to road accidents, assaults and other external causes (online supplemental figure 4). In weighted models, the hazard of all-cause mortality was 27% lower for those who attended the Vukuzazi health fairs compared with those who did not attend (HR: 0.73; (95% CI 0.65 to 0.83)) corresponding to a 1.4% reduction in mortality (table 2).

### Vukuzazi health fair attendance and clinic presentation

Overall, clinic visitation was higher among Vukuzazi health fair attenders than non-attenders both before and after the scheduled health fair. During the period between 12 months before and 12 months after the health fair, Vukuzazi attenders had on average 3440 clinic visits per month, with an increase of 450 clinic visits 1 month after the health fair while non-attenders had an average of 1500 clinic visits per month with a slight increase of 58 clinic visits 1 month after the health fair (online supplemental figure 5). There was no difference in the trend of clinic visits 12 months before and after the health fair after excluding the COVID period (online supplemental figure 6).

Among Vukuzazi attenders, we found a 13% increased odds of attending a clinic in the 3 months after versus the 3 months prior to the Vukuzazi health fairs (OR=1.13, 95% CI 1.10 to 1.17, table 3). By contrast, there was no difference in clinic attendance in the 3 months after vs prior to the health fairs in non-attenders (OR=1.02, 95% CI 0.98 to 1.06). The increased clinic attendance seen immediately after the health fairs abated in the 6 and 12 months after the health fair camp (online supplemental figure 5).

**Table 3:** Association between Vukuzazi attendance and clinic visits stratified by attendance

Any clinic visits before/after Vukuzazi	Vukuzazi non-attenders		Vukuzazi attenders	
	OR (95% CI)	P value	OR (95% CI)	P value
3 months before	Ref		Ref	
3 months after	1.13 (1.10 to 1.17)	<0.001	1.02 (0.98 to 1.06)	0.243
6 months before	Ref		Ref	
6 months after	1.04 (1.00 to 1.06)	0.007	0.95 (0.92 to 0.98)	0.001
12 months before	Ref		Ref	
12 months after	1.03 (1.00 to 1.05)	0.027	0.95 (0.93 to 0.98)	0.001

## Discussion

In a large, population-based study in rural South Africa, we found that attendance at a community-based health fair was associated with a 27% reduction in the hazard of all-cause mortality, corresponding to a 1.4% reduction in absolute mortality within 5 years of health fair attendance. This effect was most evident among those over 65 years and more evident in women than men. These findings reflect the substantial potential benefits derived from community health screening and subsequent referral to care to improve health for priority disease states in remote areas. Our findings were robust to removal of accidental deaths and trauma, adjustment for potential confounders, including age and healthcare access, and substantiated by evidence of an increase in primary healthcare clinic visitation in the 3 months following the health fairs.

Our results suggest a benefit of programmes such as health fairs that focus on free voluntary health screening in rural and historically underserved areas.^13^ We suspect, and our data support the notion, that participation in community health fairs may facilitate early detection of diseases and referrals for treatment.^29 30^ The Vukuzazi health fair specifically focused on HIV, microbiologically proven TB and NCDs, including diabetes and hypertension.^6^ It also notably included an enhanced linkage to care protocol, including an initial home visit in those with positive screening results to confirm diagnosis and promote referral to primary healthcare clinics. Thus, it is plausible that Vukuzazi health fair attendance contributed to the earlier detection of high-impact, addressable health conditions and potentially led to treatment initiation that ultimately led to a reduction in mortality, particularly for older individuals. Our results may also reflect the significance of improving access to primary healthcare. The Vukuzazi mobile health camps were strategically placed within at least two kilometres of all eligible homesteads and were located at each station for an average of 2 days. The proximity of these mobile health camps may have played a pivotal role in motivating attendance and this ease of access may have been a contributing factor to the long-term benefits of the health fair.

It is challenging to put our results into the context of prior literature because, to our knowledge, the impact of community health fairs on mortality has not been reported frequently. The Sustainable East Africa Research in Community Health (SEARCH) trial had results that were by and large similar to ours. In that study, investigators demonstrated that annual multi-disease health fairs in rural communities of Uganda and Kenya significantly improved health outcomes. Communities participating in these health fairs had earlier initiation of ART, higher levels of viral suppression among people living with HIV and better control of hypertension compared with those receiving standard care.^31^ Prior evaluations of health fairs have suggested that health fairs are linked to enhanced health knowledge and behaviour change.^14 32^ This was evident from a study in the USA which found that attendees of health fairs reported lifestyle improvement, notably in smoking cessation.^33^ Evidence from another study conducted in a semi-urban area in Nigeria demonstrates the effectiveness of health fairs in detecting various health conditions, including the rising prevalence of hypertension and obesity.^34^ Findings from a Texas study indicated a positive impact of that community health fair on community members’ health self-efficacy; however, sustained improvement in health self-efficacy was not observed.^35^ A study in Los Angeles County found that health fairs may address barriers to healthcare.^17^ Health fairs have also been demonstrated to be a successful strategy for increasing access to cancer screening in marginalised populations and have provided opportunities for vulnerable populations.^36^ Yet, few if any, studies were powered or designed like ours to measure a benefit of health fairs on all-cause mortality.

### Limitations

Our study should be considered in the context of its limitations. First and foremost is the non-randomised allocation of health fairs in our study population, which could make our study susceptible both to selection bias and confounding. For example, if some individuals were too ill to attend the health fair, that would have biased our estimates away from the null. Similarly, there is a possibility that those who attended Vukuzazi did so out of greater health-seeking behaviour, which may also be associated with improved health outcomes. We attempted to address these concerns with the use of IPTW methods, which enabled adjustment for prior healthcare visitation in our weighted models. However, there is potential for residual confounding because comorbidities were not adjusted for, as many of these conditions were assessed during the Vukuzazi health fair screening and data on pre-existing comorbidities were unavailable for non-attenders. We also note that attenders to the health fair were significantly older (with over twice as many people over 65 years of age) and so had a substantially higher expected risk of all-cause mortality. Selection bias due to health-seeking behaviour would have had to overcome the strong relationships between advanced age and all-cause mortality. We note that the study observation period included the COVID-19 pandemic period in South Africa. Thus, there is a possibility that mortality rates escalated during this period or that the pandemic impacts the generalisability of our study to non-pandemic periods. Additionally, migration differentials might have played a role, as individuals who migrated out of the study area could differ in SES, health-seeking behaviours or access to healthcare compared with those who remained, potentially introducing bias into the observed outcomes. However, we note that the HDSS does assess deaths among those who out-migrate, as long as at least one member of the household remains in the HDSS.

## Conclusions

In summary, we found that participation in a community-based health fair in rural South Africa was associated with a reduction in all-cause mortality. These findings reinforce the contribution that such programmes may have in improving and enhancing communities’ health outcomes in rural areas. We recommend consideration of such practices as part of routine healthcare delivery in similar remote areas and further exploration of their potential secondary benefits and mechanisms.

## Supplementary material

10.1136/bmjph-2025-003803 online supplemental file 1

## Data Availability

Data are available in a public, open access repository. Data are available upon reasonable request.
