# Supplementary material for: Community-based health screening and all-cause mortality in rural South Africa: a longitudinal cohort study
Source: BMJ Public Health. 2026 May 28;4(2):e003803. doi: 10.1136/bmjph-2025-003803 (PMC13223624; doi:10.1136/bmjph-2025-003803)
Supplement: online supplemental file 1 [file bmjph-4-2-s001.docx]

**Supplementary materials**

**Supplemental Figure 1: DAG: Association between Vukuzazi attendance and all-cause mortality**

**
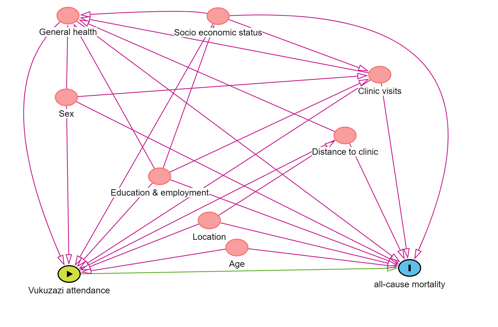
**

**Supplemental Figure 2:Distribution of the propensity score**

**
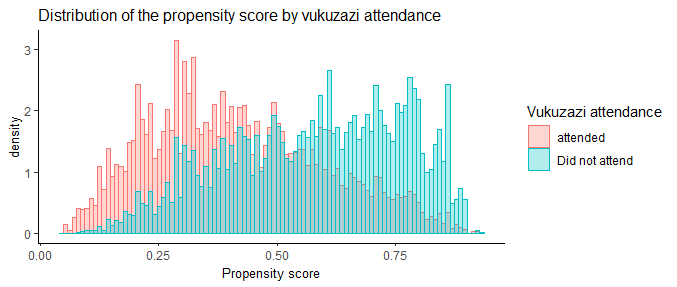
**

**Supplemental Figure 3:Covariate balance plot**


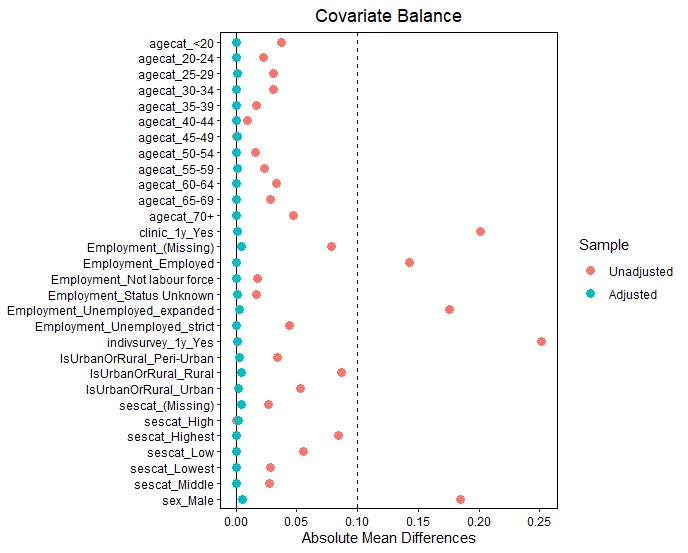


**Supplemental Figure 4: Kaplan-Meier plots showing the unweighted and weighted crude survival attenders and non-attenders**

**
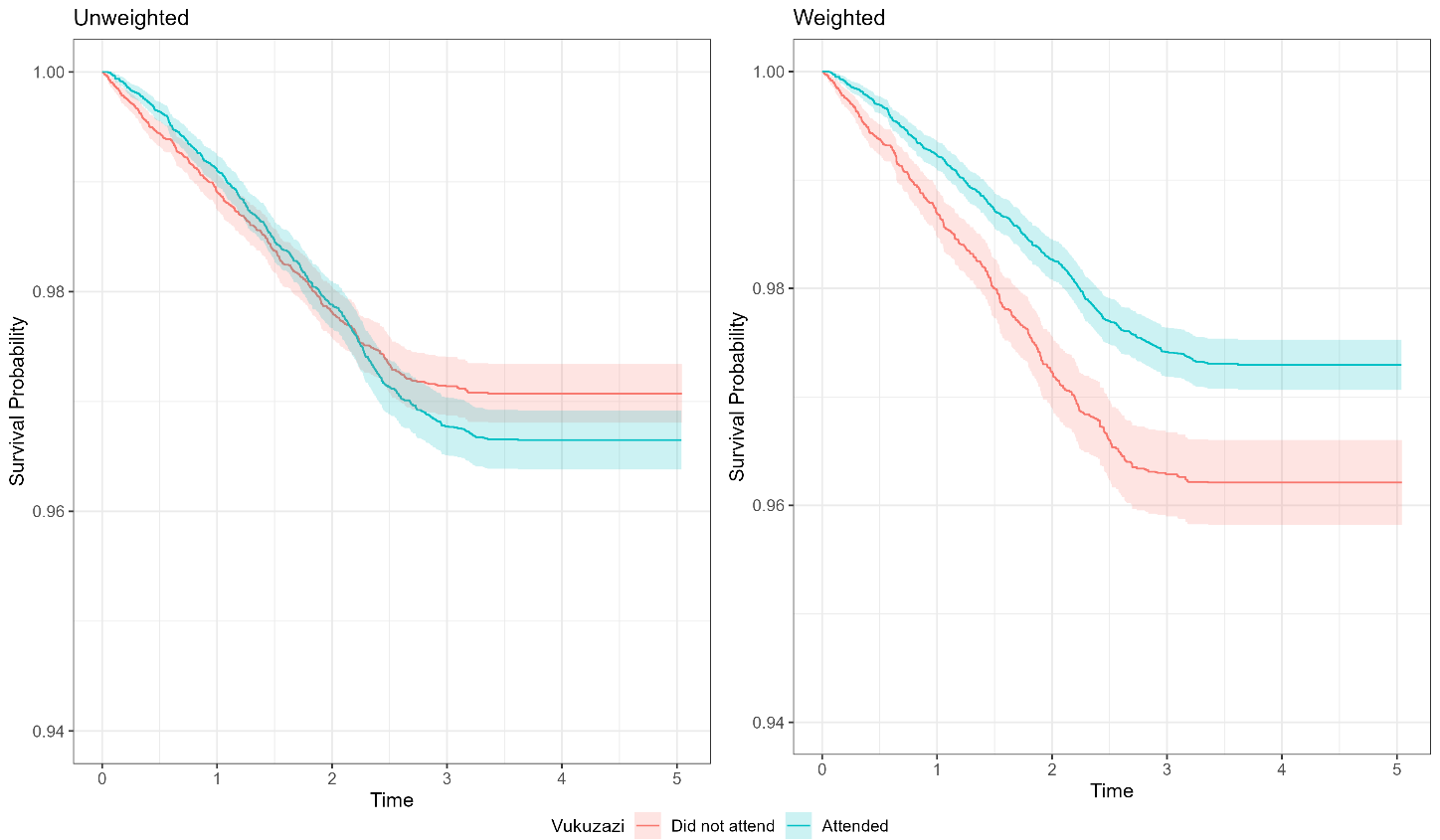
**

**Supplemental Figure 5:Clinic attendance by Vukuzazi attendance before and after Vukuzazi attendance health fair**

**
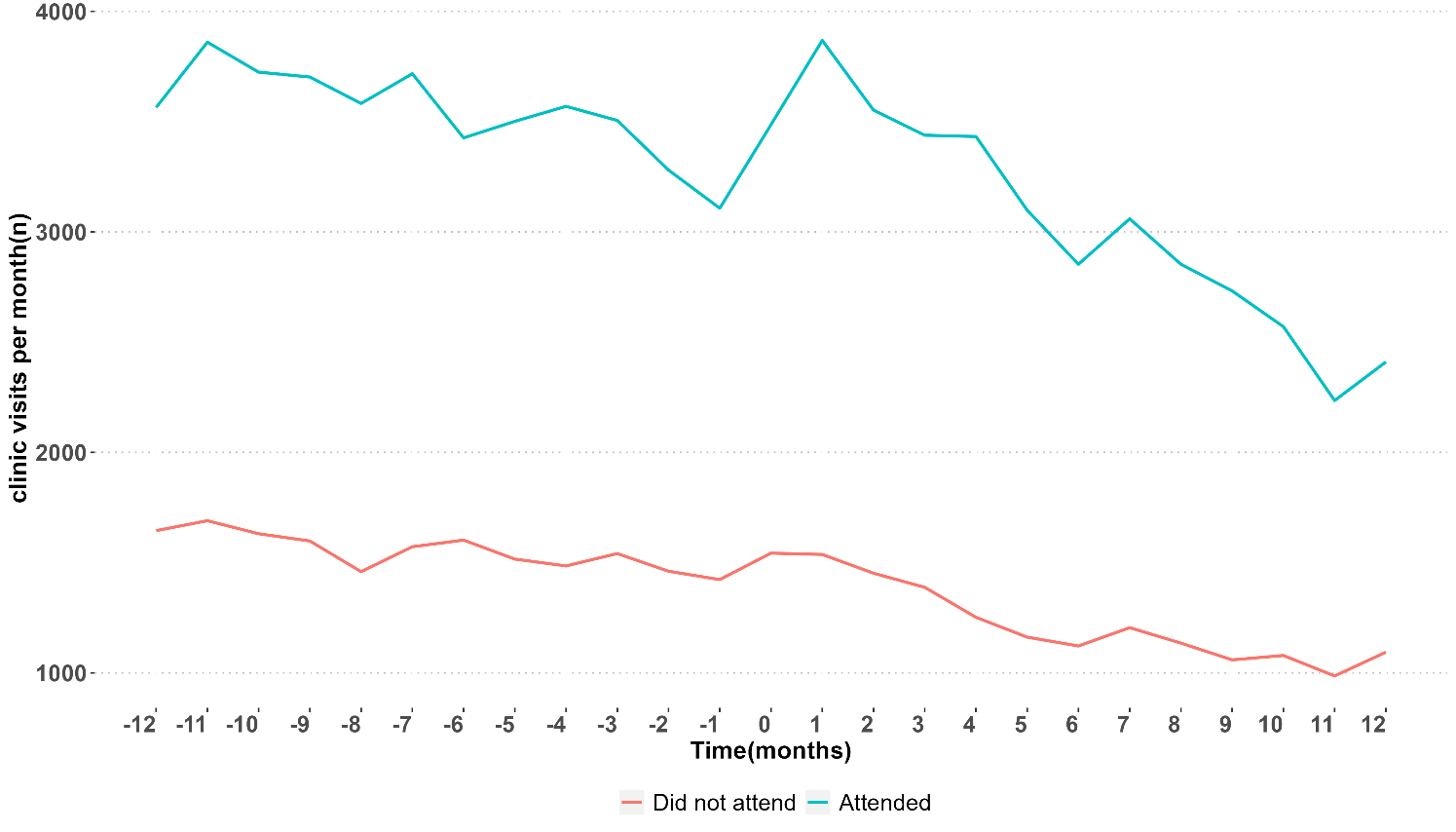
**

**Supplemental Figure 6:Clinic attendance by Vukuzazi attendance before and after Vukuzazi attendance health fair excluding the COVID period**


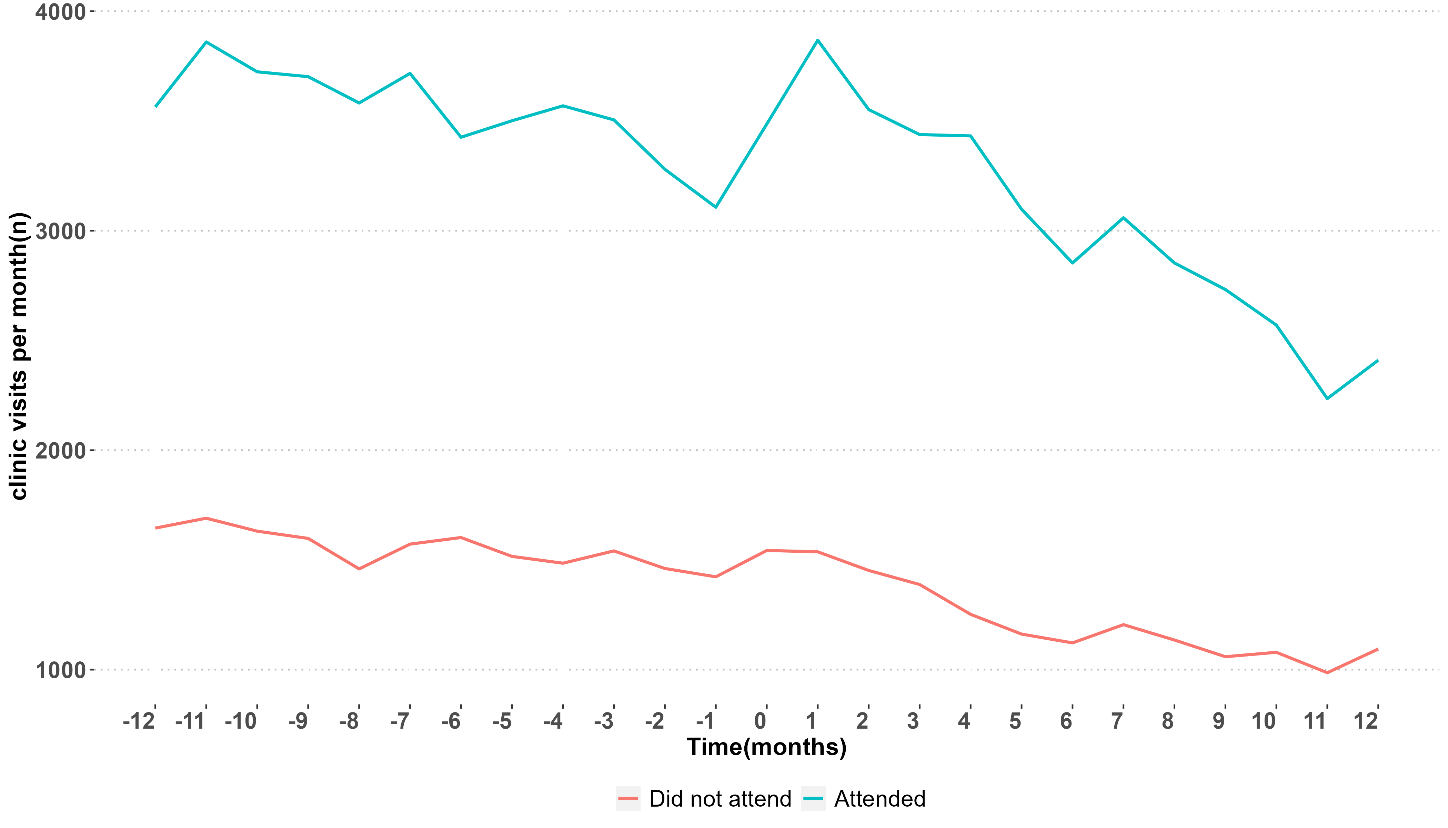


**Supplemental Table1: All-cause mortality rate by Vukuzazi attendance, sex and age group**

|  | **Total Population** | | **Vukuzazi Non-Attenders** | | **Vukuzazi Attenders** | |
| --- | --- | --- | --- | --- | --- | --- |
| **Characteristic** | **Deaths/**  **Person years** | **Rate (95%CI)*** | **Deaths/**  **Person years** | **Rate (95%CI)*** | **Deaths/**  **Person years** | **Rate (95%CI)*** |
| All Ages | 1549/127625 | 12.14  (11.54-12.76) | 675/58969 | 11.45  (10.6-12.34) | 874/68656 | 12.73  (11.9-13.6) |
| Age group |  |  |  |  |  |  |
| 15-24 | 83/39235 | 2.12  (1.68-2.62) | 44/20014 | 2.2  (1.6-2.95) | 39/19221 | 2.03  (1.44-2.77) |
| 25-44 | 285/47842 | 5.96  (5.29-6.69) | 166/24897 | 6.67  (5.69-7.76) | 119/22945 | 5.19  (4.3-6.21) |
| 45-64 | 453/28490 | 15.9  (14.47-17.43) | 176/10873 | 16.19  (13.88-18.76) | 277/17617 | 15.72  (13.93-17.69) |
| 65+ | 728/12058 | 60.38  (56.07-64.93) | 289/3185 | 90.75  (80.58-101.83) | 439/8873 | 49.48  (44.96-54.33) |

*Mortality rates are in deaths/1,000 person-years

**Supplemental Table 2: Causes of death by Vukuzazi attendance**

| **Causes of death** | **Overall**, N = 1,549^1^ | | **Did not attend**, N = 675(44%)^1^ | **Attended**, N = 874(56%)^1^ |
| --- | --- | --- | --- | --- |
|  |  | |  |  |
| **Non-communicable diseases** | | **688(44.4%)** | **282(41.8%)** | **406(46.5%)** |
| Cardiovascular diseases | | 292 (18.9%) | 104 (15.4%) | 188 (21.5%) |
| Stroke | | 115 (7.4%) | 56 (8.3%) | 59 (6.8%) |
| Cancers | | 90 (5.8%) | 41 (6.1%) | 49 (5.6%) |
| Chronic respiratory diseases | | 73 (4.7%) | 30 (4.4%) | 43 (4.9%) |
| Other non-communicable diseases | | 74 (4.8%) | 30 (4.4%) | 44 (5.0%) |
| Diabetes mellitus | | 44 (2.8%) | 21 (3.1%) | 23 (2.6%) |
|  | |  |  |  |
| **Infectious, nutritional and maternal diseases** | | **263(17.0%)** | **119(17.6%)** | **144(16.5%)** |
| HIV/AIDS related death | | 101 (6.5%) | 40 (5.9%) | 61 (7.0%) |
| Acute resp infect incl pneumonia | | 71 (4.6%) | 28 (4.1%) | 43 (4.9%) |
| Pulmonary tuberculosis | | 45 (2.9%) | 24 (3.6%) | 21 (2.4%) |
| Anemia and malnutrition | | 2 (0.1%) | 2 (0.3%) | 0 (0.0%) |
| Maternal cause of death | | 6 (0.4%) | 2 (0.3%) | 4 (0.5%) |
| Other infectious diseases | | 38 (2.5%) | 23 (3.4%) | 15 (1.7%) |
|  | |  |  |  |
| **Injuries and violent deaths** | | **108(7.0%)** | **60(8.9%)** | **48(5.5%)** |
| Other external causes | | 24 (1.5%) | 12 (1.8%) | 12 (1.4%) |
| Road accidents | | 23 (1.5%) | 12 (1.8%) | 11 (1.3%) |
| Assaults | | 61 (3.9%) | 36 (5.3%) | 25 (2.9%) |
|  | |  |  |  |
| **Indeterminate(N=76)** | | **76(4.9%)** | **46(6.8%)** | **30(3.4%)** |
| Unknown cause of death | | 76 (4.9%) | 46 (6.8%) | 30 (3.4%) |
| Missing | | 414 (26.7%) | 168 (24.9%) | 246 (28.1%) |
| ^1^n (%) *unknown cause of death | | | | |
